# Clinicopathological and prognostic factors for long-term survival in Chinese patients with metastatic renal cell carcinoma treated with sorafenib: a single-center retrospective study

**DOI:** 10.18632/oncotarget.4874

**Published:** 2015-10-14

**Authors:** Hai-Liang Zhang, Xiao-Jian Qin, Hong-Kai Wang, Wei-Jie Gu, Chun-Guang Ma, Guo-Hai Shi, Liang-Ping Zhou, Ding-Wei Ye

**Affiliations:** ^1^ Department of Urology, Fudan University, Shanghai Cancer Center, Shanghai 200032, P.R. China; ^2^ Department of Oncology, Shanghai Medical College, Fudan University, Shanghai 200032, P.R. China; ^3^ Department of Radiology, Fudan University, Shanghai Cancer Center, Shanghai 200032, P.R. China

**Keywords:** sorafenib, advanced RCC, China, overall survival, prognostic factors

## Abstract

Data on long-term survival and prognostic significance of demographic factors and adverse events (AEs) associated with sorafenib, an orally administered multikinase inhibitor in Chinese population with advanced renal cell carcinoma (RCC) are limited. Outcome data from adult patients (*n* = 256) with advanced RCC who received sorafenib (400 mg twice daily) either as first-line or second-line therapy between April 2006 and May 2013 were analyzed retrospectively. The primary endpoint was median overall survival (OS), determined to be 22.2 (95% CI: 17.1–27.4) months, and the secondary endpoint was overall median progression-free survival (PFS), determined to be 13.6 (95% CI: 10.7–16.4) months at a median follow-up time of 61.8 (95% CI: 16.2–97.4) months. Analysis of the incidence of AEs revealed the most common side effect as hand-foot skin reactions (60.5%) followed by diarrhea (38.7%), fatigue (35.5%), alopecia (34.0%), rash (24.6%), hypertension (21.5%) and gingival hemorrhage (21.1%). Multivariate regression analysis revealed older age (≥ 58 years), lower Memorial Sloan-Kettering Cancer Center score, time from nephrectomy to sorafenib treatment, number of metastatic tumors and best response as significant and independent demographic predictors for improved PFS and/or OS (*p* ≤ 0.05). Alopecia was identified as a significant and independent predictor of increased OS, whereas vomiting and weight loss were identified as significant predictors of decreased OS (*p* ≤ 0.05). Sorafenib significantly improved OS and PFS in Chinese patients with advanced RCC. Considering the identified significant prognostic demographic factors along with the advocated prognostic manageable AEs while identifying treatment strategy may help clinicians select the best treatment modality and better predict survival in these patients.

## INTRODUCTION

Renal cell carcinoma (RCC) is a heterogeneous group of tumors with distinct genetic and metabolic defects. It encompasses diverse clinical, histopathological, and molecular factors, which also have a role in differential prognosis and therapeutic responses [1]. Recent advances in the understanding of molecular biology and the cytogenetics of advanced RCC have provided unique insights into the underlying mechanisms contributing to its histological and biological diversity [2]. Consequently, targeted agents, including inhibitors of tyrosine kinase, vascular endothelial growth factor (VEGF), and mammalian target of rapamycin (mTOR) have revolutionized the therapeutic landscape in the management of patients with metastatic RCC (mRCC) [3, 4]. The efficacy of sorafenib, a multikinase inhibitor, in RCC has been previously confirmed in Phase II and Phase III trials, leading to its approval by the US Food and Drug Administration in December 2005 as the first targeted agent to show clinical activity in RCC as reviewed earlier [5]. Since then, an array of targeted drugs (tyrosine kinase inhibitors sunitinib, axitinib, and pazopanib; the VEGF monoclonal antibody bevacizumab; and the mTORs temsirolimus and everolimus) have been introduced and are now approved for clinical use [3, 6, 7]. Previous studies, including the pivotal TARGET trial, have demonstrated promising evidence for sorafenib administered at a dose of 400 mg twice daily both as a first-line and second-line therapy for advanced RCC primarily in the western population [7, 8]. These studies have shown varying improvement in progression-free survival (PFS), overall response rates, overall survival (OS), tolerance, and quality of life compared with other investigational agents including interferon, IFNα2a, tivozanib, temsirolimus, AMG 386, and axitinib [8–11].

There has been a considerable increase in the incidence of RCC and the associated mortality rates in China, which were estimated to be 2.2% and 1.2%, respectively, for all new cases of cancers excluding non-melanoma skin cancer according to GLOBOCAN worldwide estimates of cancer incidence published by the International Agency for Research on Cancer for 2012 [12]. As reviewed earlier, previous studies have shown sorafenib as a potential targeted agent with a manageable toxicity profile when used even at higher doses (1200–1600 mg/day) or in combination with other agents such as interferon, bevacizumab, temsirolimus, gemcitabine, fluorouracil, and cisplatin for treating Chinese patients with RCC [5]. Sorafenib was found to be more effective in the Chinese population when compared with the western population both as first-line and second-line treatment after failure of treatment with cytokine in patients with advanced RCC, but with a relatively higher rate of adverse events (AEs), particularly hand-foot skin reactions and alopecia. [5] However, data on the long-term survival of Chinese patients with advanced RCC treated with sorafenib are limited. The present study determined the potential of sorafenib treatment on the long-term survival in Chinese patients with metastatic RCC (mRCC) and further evaluated the prognostic factors associated with OS and/or PFS.

## RESULTS

### Baseline demographics

Between April 2006 and May 2013, a total of 317 patients with mRCC treated with sorafenib were screened at the Department of Urology, Fudan University, Shanghai Cancer Center. In all, 256 patients (age 19–89 years, median 58 years) met the inclusion criteria and were considered for the analysis. The other 61 patients were lost to follow-up. The baseline demographics and clinical characteristics are summarized in Table 1. Overall, 53.1% of the patients were aged ≥ 58 years and 71.5% were male. Almost half of the patients included in this study presented with an Memorial Sloan-Kettering Cancer Center (MSKCC) score for intermediate risk (50.8%) and the Eastern Cooperative Oncology Group (ECOG) performance status was mostly 0 (40.6%) or 1 (45.3%). The type of RCC was histologically classified as clear cell subtype in majority of the patients (78.5%), tumor nucleus grade of 3–4 (73.4%), and predominantly single tumor site (47.7%). About 79.3% of the included patients had undergone previous nephrectomy and 73.1% of the patients did not receive any previous systemic therapy.

**Table 1:** Baseline demographic and clinical characteristics of the Chinese patients with RCC treated with sorafenib

Characteristics	Number (Percentage) *N* = 256
Gender	
Male	183 (71.5%)
Female	73 (28.5%)
Age (Years)	
≥58	136 (53.1%)
<58	120 (46.9%)
BMI (Mean ± SD) (kg/m^2^)	
≥23.1	125 (48.8%)
<23.1	131 (51.2%)
MSKCC score	
Low risk	74 (28.9%)
Intermediate risk	130 (50.8%)
High risk	52 (20.3%)
ECOG performance status	
0	104 (40.6%)
1	116 (45.3%)
2	33 (12.9%)
3	3 (1.2%)
Histology	
Clear cell subtype	201 (78.5%)
Non-clear cell subtypes	55 (21.5%)
Tumor nucleus grade	
1–2	68 (26.6%)
3–4	188 (73.4%)
Previous nephrectomy	
Yes	203 (79.3%)
No	53 (20.7%)
Time from nephrectomy to sorafenib treatment Metastatic disease at diagnosis	
No palliative nephrectomy	53 (20.7%)
Palliative nephrectomy	41 (16.0%)
Metastatic disease after radical nephrectomy	
≥12 months	98 (38.3%)
<12 months	64 (25.0%)
Metastatic organs	
1	122 (47.7%)
2	99 (38.7%)
3	29 (11.3%)
4	6 (2.3%)
Previous systemic therapy	
None	187 (73.1%)
Cytokine	60 (23.4%)
Sunitinib	9 (3.5%)

BMI, body mass index; ECOG, Eastern Cooperative Oncology Group; MSKCC, Memorial Sloan-Kettering Cancer Center; OS, overall survival; PFS, progression-free survival; RCC, renal cell carcinoma.

### Best tumor response

**Figure 1:**
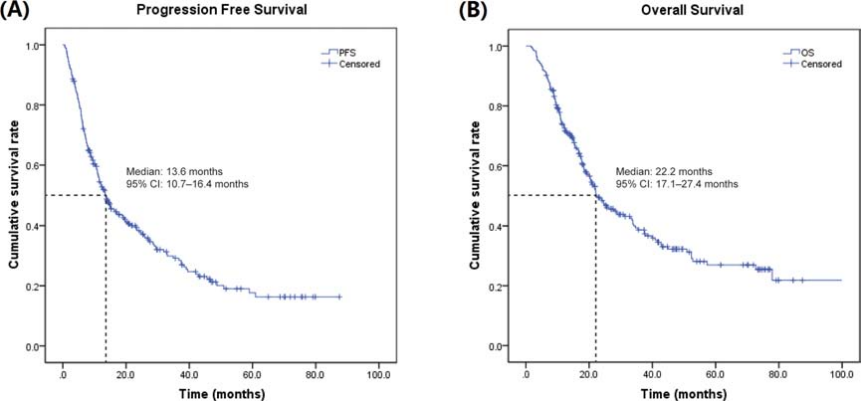
Kaplan-Meir estimates of PFS and OS in Chinese patients with mRCC treated with sorafenib mRCC, metastatic renal cell carcinoma; OS, overall survival; PFS, progression free survival.

Best tumor response was primarily characterized as complete remission (CR), partial remission (PR), stable disease (SD), and progressive disease (PD), respectively, in 2 (0.8%), 45 (17.6%), 176 (68.7%), and 33 (12.9%) of the included patients. Clinical benefit rate (CR, PR, and SD) was found to be 87.1%, while object response rate (CR and PR) was found to be 18.4%. Between the first sorafenib treatment and the study cut-off date (May 31, 2014), a total of 176 patients reached PD and 153 patients died after a median follow-up time of 61.8 (16.2–97.4) months. The Kaplan-Meier estimates for PFS and OS are presented in Figure 1A and Figure 1B respectively. Overall median PFS was 13.6 (95% CI: 10.7–16.4) months and the median OS was 22.2 (95% CI: 17.1–27.4) months.

### Prognostic factors for survival

**Figure 2:**
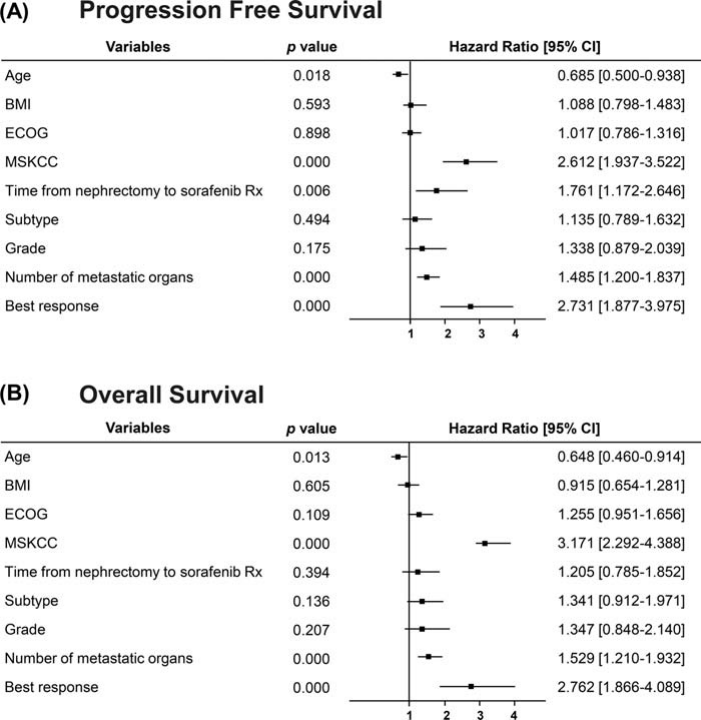
Forest plots displaying multivariate Cox analysis of demographic variables prognostic to PFS and OS in Chinese patients with mRCC treated with sorafenib BMI, body mass index; CI, confidence interval; ECOG, Eastern Cooperative Oncology Group; mRCC, metastatic renal cell carcinoma; MSKCC, Memorial Sloan-Kettering Cancer Center; OS, overall survival; PFS, progression free survival.

Univariate analysis was used to screen for potential prognostic factors such as gender, age, body mass index (BMI), MSKCC score, ECOG performance status, histology, tumor nucleus grade, previous nephrectomy, time from nephrectomy to sorafenib treatment, number of metastatic organs, previous systemic therapy, and best response (Table 2). The probable prognostic factors with a *p* < 0.1 including age, BMI, ECOG performance status, MSKCC score, time from nephrectomy to sorafenib treatment, histology, tumor nucleus grade, number of metastatic organs, and best response were entered into the multivariate Cox proportional hazard regression model for analysis. Older age, lower MSKCC score, lower number of metastatic organs, and best tumor response were found to be significant and independent predictors for improved PFS and OS. Additionally, time from nephrectomy to sorafenib treatment also significantly predicted improved PFS but not OS (*p* ≤ 0.05; Figure 2A and 2B).

**Table 2:** Baseline prognostic factors of PFS and OS in Chinese patients with mRCC treated with sorafenib

Characteristics	Median PFS (95% CI) (Months)	*p* value	Median OS (95% CI) (Months)	*p* value
Gender				
Male	14.3 (9.7, 18.9)	0.163	23.4 (14.0, 32.8)	0.368
Female	11.1 (9.7, 12.4)		22.2 (14.4, 30.1)	
Age (Years)				
≥58	23.3 (13.9, 32.7)	0.020	33.8 (22.7, 44.9)	0.082
<58	10.9 (9.0, 12.8)		20.4 (16.9, 24.0)	
BMI (Mean ± SD) (kg/m^2^)				
≥23.1	14.1 (11.2, 17.5)	0.263	33.6 (22.0, 45.2)	0.050
<23.1	11.5 (6.7, 16.0)		21.1 (18.1, 25.6)	
MSKCC score				
Low risk	42.1 (17.8, 66.3)	<0.001	97.2 (NA, NA)	<0.001
Intermediate risk	11.6 (8.6, 14.7)		23.4 (20.2, 26.6)	
High risk	5.1 (3.4, 6.8)		8.8 (6.9, 10.7)	
ECOG performance status				
0	25.7 (17.1, 34.3)	<0.001	44.9 (29.4, 60.4)	<0.001
1	11.5 (8.5, 14.5)		20.7 (17.6, 23.7)	
2	5.9 (0.7, 11.1)		10.8 (8.1, 13.5)	
3	3.4 (2.3, 4.4)		8.8 (6.5, 11.0)	
Histology				
Clear cell subtype	16.3 (10.0, 22.6)	0.001	28.7 (17.7, 37.7)	<0.001
Non-clear cell subtypes	6.9 (5.9, 8.2)		11.3 (4.7, 17.8)	
Tumor nucleus grade				
1–2	35.0 (24.2, 45.8)	<0.001	52.4 (26.5, 78.2)	<0.001
3–4	10.8 (9.0, 12.7)		19.4 (16.2, 22.6)	
Previous nephrectomy				
Yes	13.6 (7.8, 19.4)	0.800	24.1 (15.5, 32.7)	0.041
No	13.4 (10.0, 16.8)		18.4 (11.5, 25.3)	
Time from nephrectomy to sorafenib treatment				
Metastatic disease at diagnosis				
No palliative nephrectomy	13.8 (7.9, 19.7)	<0.001	18.4 (10.3, 26.5)	<0.001
Palliative nephrectomy	11.3 (5.5, 17.0)		23.4 (9.8, 36.9)	
Metastatic disease after radical nephrectomy				
≥12 months	27.3 (15.2, 39.5)		77.9 (30.1, 125.7)	
<12 months	7.1 (5.5, 8.7)		16.8 (12.1, 21.5)	
Metastatic organs				
1	28.8 (20.8, 36.9)	<0.001	42.7 (27.1, 58.2)	<0.001
2	10.5 (7.5, 13.6)		18.7 (15.7, 21.7)	
3	7.5 (3.3, 11.7)		11.1 (7.0, 15.1)	
4	4.0 (2.9, 5.2)		5.4 (3.4, 7.4)	
Previous systemic therapy				
None	14.1 (4.6, 23.5)	0.109	24.9 (17.4, 32.4)	0.246
Cytokine	13.6 (10.3, 16.8)		22.4 (16.2, 28.7)	
Sunitinib	6.3 (3.5, 9.1)		11.3 (9.6, 13.0)	
Best response				
CR	46.7 (NA, NA)	<0.001	Not reached	<0.001
PR	25.0 (17.8, 32.2)		42.7 (26.5, 59.7)	
SD	13.7 (10.1, 17.3)		22.4 (19.3, 22.7)	
PD	2.1 (1.7, 2.5)		6.7 (5.4, 7.9)	

BMI, body mass index; CR, complete remission; ECOG, Eastern Cooperative Oncology Group; MSKCC, Memorial Sloan-Kettering Cancer Center; NA, not applicable as was not achieved; OS, overall survival; PD, disease progression; PFS, progression-free survival; PR, partial remission; SD, stable disease.

### Adverse events

**Figure 3:**
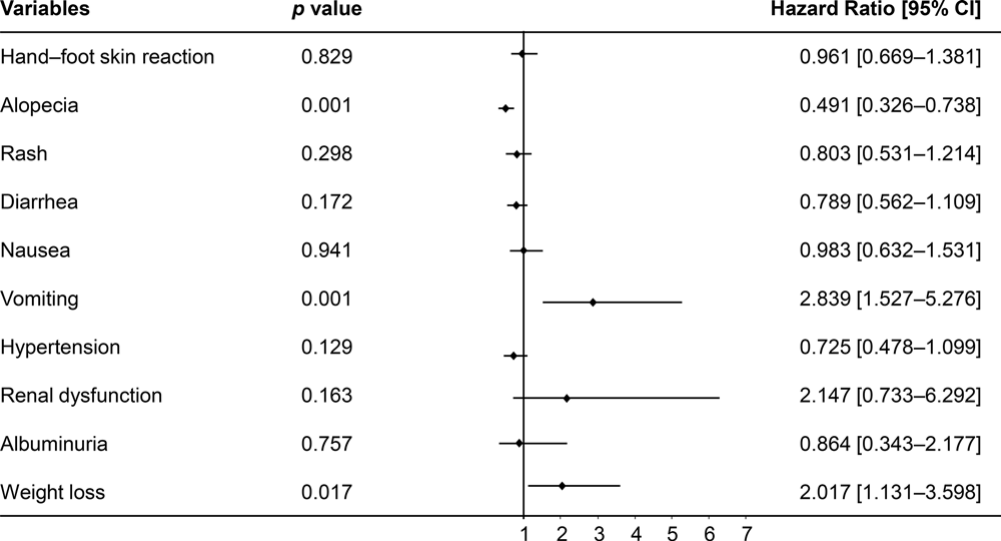
Forest plots displaying multivariate Cox analysis of AEs prognostic to OS in Chinese patients with mRCC treated with sorafenib AEs, adverse events; BMI, body mass index; CI, confidence interval; ECOG, Eastern Cooperative Oncology Group; mRCC, metastatic renal cell carcinoma; MSKCC, Memorial Sloan-Kettering Cancer Center; OS, overall survival.

Analysis of AEs in the included patients revealed the most common side effect as hand-foot skin reactions (60.5%) followed by diarrhea (38.7%), fatigue (35.5%), alopecia (34.0%), rash (24.6%), hypertension (21.5%), and gingival hemorrhage (21.1%). The AEs recorded in >1% of the included patients during the study period and their grades are listed in Table 3. Univariate analysis revealed the incidence of most of the commonly encountered AEs such as hand-foot skin reactions, alopecia, rash, diarrhea, and hypertension to be associated with increased OS, while other AEs such as vomiting, albuminuria, and weight loss were associated with decreased OS (*p* ≤ 0.05) (Table 4). The probable prognostic factors with a *p* < 0.1 were entered into the multivariate Cox proportional regression model for analysis. Multivariate analysis revealed alopecia to be a significant and independent predictor for increased OS, whereas vomiting and weight loss were identified as significant predictors for decreased OS (*p* ≤ 0.05) (Figure 3).

**Table 3:** Summary of common AEs with an incidence of ≥ 1% for all grades

Adverse events	All grades *n* (%)	Grade 2 *n* (%)	Grades 3–4 *n* (%)
Hand-foot skin reaction	155 (60.5)	65 (25.4)	21 (8.2)
Alopecia	87 (34.0)	20 (7.8)	0 (0.0)
Rash	63 (24.6)	25 (9.8)	3 (1.2)
Diarrhea	99 (38.7)	51 (19.9)	9 (3.5)
Constipation	33 (12.9)	7 (2.7)	0 (0.0)
Nausea	49 (19.1)	17 (6.6)	1 (0.4)
Vomiting	19 (7.4)	5 (2.0)	1 (0.4)
Hypertension	55 (21.5)	23 (9.0)	2 (0.8)
Angina pectoris and myocardial infarction	3 (1.2)	2 (0.8)	1 (0.4)
Anemia	41 (16.0)	17 (6.6)	4 (1.6)
Leukopenia	7 (2.7)	1 (0.4)	0 (0.0)
Thrombocytopenia	4 (1.6)	1 (0.4)	0 (0.0)
Mucositis	46 (18.0)	19 (7.4)	7 (2.7)
Liver dysfunction	39 (15.2)	17 (6.6)	11 (4.3)
Renal dysfunction	18 (7.0)	3 (1.2)	1 (0.4)
Albuminuria	28 (10.9)	11 (4.3)	5 (2.0)
Gingival hemorrhage	54 (21.1)	12 (4.7)	0 (0.0)
Stool hemorrhage	43 (16.8)	11 (4.3)	2 (0.8)
Hemoptysis	27 (10.5)	8 (3.1)	3 (1.2)
Fatigue	91 (35.5)	32 (12.5)	11 (4.3)
Weight loss	23 (9.0)	12 (4.7)	1 (0.4)

AEs, adverse events.

**Table 4:** Prognostic implication of AEs on OS in Chinese patients with mRCC treated with sorafenib

Adverse events	Number of patients	Median OS (95% CI) (Months)	*p* value
Hand-foot skin reaction			
Yes	155	25.5 (16.0, 35.1)	0.028
No	101	16.7 (11.8, 21.7)	
Alopecia			
Yes	87	41.5 (25.1, 58.0)	< 0.001
No	169	18.4 (15.2, 21.6)	
Rash			
Yes	63	37.7 (17.2, 58.2)	0.018
No	193	20.9 (18.0, 23.9)	
Diarrhea			
Yes	99	28.7 (17.9, 39.5)	0.047
No	157	20.1 (13.4, 26.8)	
Constipation			
Yes	33	22.8 (15.7, 29.8)	0.887
No	223	22.1 (20.2, 24.1)	
Nausea			
Yes	49	20.1 (18.4, 25.0)	0.083
No	207	23.7 (18.7, 30.4)	
Vomiting			
Yes	19	15.2 (11.1, 19.3)	0.011
No	237	24.7 (17.4, 31.8)	
Hypertension			
Yes	55	40.1 (28.3, 53.5)	0.019
No	201	21.1 (17.9, 24.2)	
Angina pectoris and myocardial infarction			
Yes	3	3.6 (1.3, 12.8)	0.217
No	253	22.3 (18.2, 26.5)	
Anemia			
Yes	41	19.9 (17.9, 24.2)	0.126
No	215	23.7 (18.9, 28.8)	
Leukopenia			
Yes	7	21.9 (18.2, 26.5)	0.773
No	249	22.6 (18.4, 27.3)	
Thrombocytopenia			
Yes	4	33.3 (17.3, 55.8)	0.527
No	252	22.1 (18.5, 25.5)	
Mucositis			
Yes	46	25.5 (9.8, 41.3)	0.214
No	210	21.9 (16.1, 27.6)	
Liver dysfunction			
Yes	39	31.1 (16.8, 45.5)	0.109
No	217	22.0 (18.3, 25.7)	
Renal dysfunction			
Yes	18	16.9 (9.4, 24.5)	0.027
No	238	24.6 (17.3, 32.0)	
Albuminuria			
Yes	28	18.4 (15.5, 21.3)	0.046
No	228	24.6 (19.9, 29.4)	
Gingival hemorrhage			
Yes	54	24.2 (16.9, 31.6)	0.173
No	202	21.7 (16.9, 26.3)	
Stool hemorrhage			
Yes	43	22.9 (16.7, 29.9)	0.775
No	213	22.3 (18.5, 26.1)	
Hemoptysis			
Yes	27	22.1 (12.8, 31.2)	0.459
No	229	23.7 (18.8, 28.7)	
Fatigue			
Yes	91	20.8 (15.9, 25.8)	0.108
No	165	25.1 (18.4, 31.3)	
Weight loss			
Yes	23	17.5 (14.9, 20.1)	0.027
No	233	24.9 (16.9, 32.9)	

AEs, adverse events; mRCC; metastatic renal cell carcinoma; OS, overall survival.

## DISCUSSION

The efficacy and tolerability of sorafenib in the treatment of advanced RCC is well established in the global population, while the data on long-term survival are primarily from the western population [5, 7, 8, 13–21]. Long-term survival rates following sorafenib treatment are, however, limited in patients of Asian origin, particularly China, wherein there is an increasing incidence of RCC contributing to increased mortality and reduced survival. The present retrospective study contributes valuable insights into the long-term survival of Chinese patients with advanced RCC treated with sorafenib as first-line or second-line therapy.

The findings from our study suggest that sorafenib is effective in improving the OS and PFS in Chinese patients with advanced RCC both as first-line and second-line treatment, with a manageable toxicity profile. Median OS was found to be 22.2 months in our study, which seemed to be higher than that reported in earlier studies in the Chinese and western populations (11.7–17.8 months). Median PFS (13.6 months) and clinical benefit rate ([CR+PR+SD], 87.1%) were also found to be consistent with the previous reports from China (PFS, 9.6–15 months; clinical benefit rate, 80%–88%) and appeared to be greatly improved than those reported in western populations (PFS, 5.5–6.6 months; clinical benefit rate, 84%–85%). The objective response rate ([CR+PR], 18.4%) was similar or lower than that reported in previous studies from China (16.7%–36.6%), but was higher than the reports from the western populations (4.0%–10.2%) [5, 13–20]. However, the baseline demographics and clinical characteristics of the patients included in this investigation were different from the previous western studies [8–11]. The observed improvements in survival, therefore, need to be further validated in future trials or propensity matched studies comparing the efficacy of sorafenib between Chinese patients and Western populations. Ethnic differences were earlier demonstrated to influence incidence rates and survival rates in RCC [22]. Overall, the better clinical outcomes of sorafenib treatment in Chinese patients with advanced RCC compared with patients of western origin are probably due to the difference in ethnicity and the associated differences in molecular features as reviewed earlier [5]. Polymorphism of cancer susceptibility genes, which in turn may be associated with the ethnicity, was earlier suggested as a potential predictor of outcome and toxicity of tyrosine kinase inhibitors including sorafenib [5, 23, 24].

The multivariate analysis of prognostic demographic factors revealed older age (≥ 58 years) to be a significant predictor of improved PFS and OS in patients with metastatic RCC treated with sorafenib (*p* ≤ 0.05). Sorafenib was thus confirmed to be effective in elderly patients, as illustrated in earlier studies [8, 25]. The baseline MSKCC score was also found to be a significant and strong predictor for both PFS and OS. Lower scores predicted improved PFS and OS, while higher scores predicted decreased PFS and OS, suggesting poorer prognosis. Tanigawa et al. [26] also reported favorable prognosis according to the MSKCC risk groups to be a significant and strong factor for predicting superior PFS in patients with advanced RCC on sorafenib treatment from Japan. This observation was earlier attributed to the higher levels of VEGF in patients with higher MSKCC scores compared with patients with lower scores at baseline [9]. The involvement of multiple organs in mRCC in our study significantly predicted reduced PFS and OS, as reported earlier [27]. In addition, the best tumor response was also identified as a significant predictor for improved PFS and OS in the order of CR > PR > SD > PD (*p* < 0.05). Furthermore, the time from nephrectomy to sorafenib treatment was also found to be a significant predictor of improved PFS and OS. There was no statistically significant difference in PFS as well as OS when sorafenib is administered as the first-line or second-line treatment after cytokine or sunitinib therapy for mRCC. These results suggest similar benefits of sorafenib in first- and second-line patients as demonstrated earlier in a nonrandomized, open-access trial [16]. AEs were not compared between lines of sorafenib treatment in our study. Expert opinion based on available evidence however suggests similar incidence of AEs in first-line and subsequent lines of sorafenib therapy in patients with mRCC [28].

Although not significant in multivariate regression model, univariate analysis of prognostic demographic variables in our study also indicated high BMI (≥ 23.1 kg/m^2^), lower ECOG performance status, clear cell subtype of RCC, lower tumor nucleus grade and time from nephrectomy to sorafenib treatment to be associated with better outcomes including PFS, and/or OS with sorafenib treatment in Chinese patients. Previous studies also reported an association of pre-operative obesity, a cause of RCC, with better prognosis and improved OS in patients with RCC [29, 30]. Several explanations for this were reviewed by these authors, including increased fat between the kidney and Gerota's fascia in obese patients acting as a barrier to further invasion of cancer cells and better nutritional status contributing to improved survival, while cachexia associated with underweight patients predicted poorer prognosis [29, 30]. This observation is further strengthened by our recent report wherein higher visceral adiposity, a comprehensive indicator of nutritional status and biological factors, was found to correlate strongly with reduced mortality in the patients who received VEGF-targeted therapy [31]. Treatment with sorafenib as an anti-angiogenic agent was earlier suggested to be less effective for non-clear cell subtypes of RCC [32]. As reviewed earlier, deregulated VEGF or mTOR pathways associated with the inactivation of the von Hippel Lindau (VHL) gene are important therapeutic targets in clear cell RCC. Improved clinical outcomes with targeted agents including sorafenib were, therefore, advocated in patients with RCC of the clear cell subtype but not the non-clear cell subtype [33]. Lower grade (grade 1–2) of tumors based on necrosis, illustrated to be associated with improved PFS and OS in our study, was earlier reported as an independent predictor [34].

The AE profile in our study, compared with data from patients of western origin reported in the TARGET study, revealed a higher incidence of hand-foot skin reaction (60.5% vs. 30%), alopecia (34% vs. 27%), hypertension (21.5% vs. 17%), and anemia (16% vs. 8%). However, these results are consistent with the earlier reports on the tolerability of sorafenib in Chinese patients with advanced RCC, as well as in patients with hepatocellular carcinoma in the Asian-Pacific trial [5, 14, 15, 17–20, 35]. This observation may again be attributed to the difference in ethnicity and the associated differences in molecular features as explained earlier. There were also events of bleeding, liver dysfunction, and angina similar to reports from earlier studies in Chinese patients with advanced RCC treated with sorafenib [5, 15, 20]. Univariate analysis of the prognostic significance of AEs encountered in this study with the median OS revealed a significant association of hand-foot skin reaction, alopecia, rash, diarrhea, and hypertension towards improved median OS in Chinese patients treated with sorafenib (*p* ≤ 0.05). Multivariate analysis, however, revealed alopecia to be a significant, independent and strong predictor for improved OS, while vomiting and weight loss were identified as significant predictors for decreased OS. The higher incidence of AEs related to skin reactions and its association with the OS corroborate the findings from earlier studies including pooled safety analyses of sorafenib in the treatment of solid tumors, including RCC, which have demonstrated a significant association of the severity of the skin reactions with the time to progression [5, 36–39]. A single-center retrospective study from Japan suggested that sorafenib induced hand-foot skin reaction was associated with improved best tumor response, and PFS was a useful biomarker of clinical outcome in patients with mRCC [40]. Although continuous dosing of sorafenib in patients with advanced RCC was earlier suggested to be associated with an increased incidence of diarrhea, its prognostic significance related to survival in these patients is lacking [20, 41]. Diarrhea was, however, suggested to be an independent positive prognostic factor for prolonged OS in patients with hepatocellular carcinoma treated with sorafenib [37, 42, 43]. Our study reported a high incidence of hypertension, mainly Grade 1 or Grade 2. This observation was similar to a previous study that reported a higher incidence of low-grade sorafenib-induced hypertension in Chinese patients with advanced RCC that was managed effectively with antihypertensive therapy, with no reports of associated cardiac events, hypertensive stress, dose reduction, or treatment discontinuation. Sorafenib-induced hypertension was associated with improved median OS (*p* ≤ 0.02), although it was not a significant predictor of OS in our study as reported earlier [44]. A systematic review and meta-analysis of studies in cancer patients treated with sorafenib revealed a significantly higher incidence of hypertension in patients with RCC, and the occurrence was further suggested to be associated with improved prognosis [45]. AEs such as vomiting, albuminuria, and weight loss were, however, found to be associated with significantly reduced median OS in our study (*p* ≤ 0.05). Anorexia with or without nausea, vomiting, or diarrhea related to targeted therapies for RCC, presented as dramatic weight loss, was associated with deteriorated quality of life and survival. Albuminuria ≤ Grade 2 was reported to be common in patients treated with VEGF inhibition, but is rarely ≥ Grade 3 or nephrotic and is suggested to be associated with renal dysfunction and/or hypertension, which in turn may affect OS. Overall, the potential emergence of certain low-grade AEs such as hand-foot skin reactions and hypertension is, therefore, advocated in patients with advanced RCC treated with sorafenib to be associated with improved survival [6, 46–49].

The retrospective design and the sample size of this study may be inadequate to comprehensively determine the prognostic significance of sorafenib on OS in Chinese patients with mRCC.

This study provides a valuable insight into the long-term safety and efficacy of sorafenib as a first-line or second-line therapy to treat advanced RCC in patients from China. The identified significant prognostic baseline predictors for improved clinical outcomes with respect to PFS and/or OS were higher age, low MSKCC score, time from nephrectomy to sorafenib treatment, number of metastatic tumors, and best response. Alopecia, an AE of prognostic significance, was identified as an independent and strong predictor for improved OS, whereas vomiting and weight loss were significant predictors for decreased OS. The identified prognostic factors and AEs will be helpful in establishing realistic patient expectations and in guiding treatment decisions to help the clinicians employ the most suitable treatment strategy involving sorafenib in order to improve the survival in these patients.

## MATERIALS AND METHODS

This retrospective study was approved by the institutional review board of Fudan University, Shanghai Cancer Center, Shanghai, China. Consecutive patients with mRCC who provided informed consent and were treated with sorafenib were screened in the Department of Urology, Fudan University, Shanghai Cancer Center between April 2006 and May 2013 for inclusion in this study. Adult patients with follow-up data available for at least 1 year with baseline computed tomography (CT) scans that revealed at least one measurable metastatic lesion (≥ 10 mm in greatest diameter) and at least one follow-up CT scan after treatment assessed as per the Response Evaluation Criteria In Solid Tumors (RECIST) criteria were included for analysis. Patients who were lost to follow-up were excluded from this study. Patient records were retrospectively reviewed and the CT scans were reviewed independently by a senior radiologist.

### Treatment and follow-up

Patients were administered sorafenib either as first-line or second-line treatment for mRCC. All patients received oral sorafenib 400 mg twice daily on a continuous dosing schedule, at an interval of 12 hours. Treatment was continued until disease progression or unacceptable toxicity. Dose reduction to 400 mg once daily was allowed for unacceptable toxicities including Grade 3 or 4 hematological toxicity, skin toxicity, hypertension, and/or hepatic dysfunction as defined by the National Cancer Institute's Common Terminology Criteria for Adverse Events (NCI-CTCAE) version 4.0 at the discretion of the attending urologists.

Follow-ups were scheduled for all patients every month during their treatment with sorafenib or every 3 months after the discontinuation of their treatment in case of unacceptable toxicity. Assessments included protocol-mandated evaluation of complete history, physical examination, and routine laboratory tests (complete blood count, serum electrolytes, and liver and renal function tests). Tumor changes were assessed with a CT scan starting at 6 weeks after the initiation of the treatment using the best response as per the RECIST criteria and PFS. Follow-up RECIST measurements were ordered every 6–8 weeks during the treatment and at every follow-up visit after the termination of the treatment.

### Outcomes and assessments

The primary end point was OS (calculated from the date of the first dose of sorafenib to the date of death or last follow-up) and the secondary end point was PFS (time from the first administration of sorafenib to the first documentation of disease progression or death from any cause). The safety outcome measures were incidence of AEs from the first administration of sorafenib to the last follow-up. Exploratory analysis included evaluation of the effect of important prognostic factors such as age, gender, MSKCC score, ECOG performance, previous nephrectomy, previous systemic therapy and number of metastatic tumors with PFS and OS, and the association of AEs with OS.

### Statistical analysis

The statistical analyses of the collected data were performed using SPSS software version 19. Continuous variables such as PFS and OS were reported as medians and interquartile ranges, and categorical data such as age, gender, previous nephrectomy, or systemic therapy were presented as proportions. The follow-up duration was calculated using the reversed Kaplan-Meier method. The Shapiro-Wilk test was used to evaluate the data for normality distribution. OS and PFS were estimated using the Kaplan-Meier method with Rothman's 95% CI and compared across the groups using the log-rank test. The Cox proportional hazards model was used to evaluate the prognostic value of the investigated parameters. All *p* values were two-sided and were considered significant if *p* value was < 0.05. The concordance index and the proportion of 2 variance explained (R) were computed to assess the prediction performance for survival (PFS, OS).
